# Emergence of a KPC Variant Conferring Resistance to Ceftazidime-Avibactam in a Widespread ST11 Carbapenem-Resistant *Klebsiella pneumoniae* Clone in China

**DOI:** 10.3389/fmicb.2021.724272

**Published:** 2021-08-16

**Authors:** Xi Li, Jingjing Quan, Huanhuan Ke, Wenhao Wu, Yu Feng, Yunsong Yu, Yan Jiang

**Affiliations:** ^1^Centre of Laboratory Medicine, Zhejiang Provincial People’s Hospital, People’s Hospital of Hangzhou Medical College, Hangzhou, China; ^2^Department of Infectious Diseases, Sir Run Run Shaw Hospital, Zhejiang University School of Medicine, Hangzhou, China; ^3^Regional Medical Center for National Institute of Respiratory Diseases, Sir Run Run Shaw Hospital, Zhejiang University School of Medicine, Hangzhou, China; ^4^Key Laboratory of Microbial Technology and Bioinformatics of Zhejiang Province, Hangzhou, China; ^5^Department of Biophysics, Zhejiang University School of Medicine, Hangzhou, China; ^6^Department of Pathology, Sir Run Run Shaw Hospital, Zhejiang University School of Medicine, Hangzhou, China

**Keywords:** ceftazidime-avibactam, resistance, CRKP, KPC-74, ST11

## Abstract

Carbapenem-resistant *Klebsiella pneumoniae* (CRKP) infection poses a great threat to public health worldwide, and KPC-2-producing strains are the main factors responsible for resistance to carbapenems in China. Ceftazidime/avibactam (CZA) is a novel β-lactam/β-lactamase inhibitor combination with good activity against KPC-2 carbapenemase and is becoming the most important option for treating KPC-producing CRKP infection. Here, we report the emergence of a novel KPC-2 variant, designated KPC-74, produced by *K. pneumoniae* strain KP55, that conferred CZA resistance in a patient after CZA exposure. The novel *bla*_KPC–74_ variant showed a deletion of 6 nucleotides at positions 712–717 compared with *bla*_KPC–2_, and this deletion resulted in the consequent deletion of glycine and valine at positions 239 and 240. Antimicrobial susceptibility testing showed that KP55 presents multidrug resistance, including resistance to CZA and ertapenem, but is susceptible to imipenem, meropenem, and colistin. The *bla*_KPC–74_ gene was located on a plasmid, as determined by S1-nuclease pulsed-field gel electrophoresis followed by southern blotting, and confirmed to be 133,766 bp in length by whole-genome sequencing on both the Illumina and MinION platforms. The CZA resistance phenotype of the novel KPC variant was confirmed by both transformation of the *bla*_KPC–74_-harboring plasmid and a *bla*_KPC–74_ gene cloning assay, showing a 64-fold higher CZA minimum inhibitory concentration (MIC) than the recipient strains. The G239_V240del observed in KPC-74 was outside the omega-loop region but was still close to the active site Ser70 and omega-loop in the protein tertiary structure. The enzyme kinetic parameters and IC_50_ values further indicated that the hydrolytic activity of the KPC-74 enzyme against ceftazidime was potentiated twofold and that the affinity between KPC-74 and avibactam was alleviated 17-fold compared with that of the KPC-2 allele. This CZA resistance mediated by KPC-74 could be selected after CZA therapy and evolved to be more diverse and heterogeneous. Surveillance of CZA resistance is urgently needed in clinical settings.

## Introduction

Gram-negative bacteria producing carbapenem-hydrolysing β-lactamase enzymes (carbapenemases) are becoming increasingly prevalent worldwide and have become one of the major threats to public health (An et al., 2018). Among these carbapenemases, *Klebsiella pneumoniae* carbapenemase (KPC) is the most common serine A type β-lactamase; this enzyme mediates high-level resistance to carbapenems and is usually carried by *K. pneumoniae* (Tzouvelekis et al., 2012). Carbapenem-resistant *K. pneumoniae* (CRKP) infection is commonly associated with high morbidity and mortality rates and thus poses a severe challenge in clinical treatment (Zhang et al., 2018). In China, KPC-2 is the main factor responsible for the resistance to carbapenems, and the vast majority of KPC-2-producing clinical CRKP isolates belong to sequence type (ST) 11 based on several epidemiological and surveillance studies (Zhang et al., 2017, 2018; Wang et al., 2018). Infections caused by these endemic ST11 KPC-2-producing CRKP isolates are usually difficult to treat and greatly limit therapeutic options.

Ceftazidime/avibactam (CZA) is a novel β-lactam/β-lactamase inhibitor combination available in China since 2019 with activity against Ambler class A (including KPCs), class C and some class D β-lactamases but is ineffective against class B metallo-β-lactamases (Falcone and Paterson, 2016). CZA has become an important first-line option for treating KPC-producing CRKP infection. However, although CZA was only recently introduced in clinical practice and the general resistance rates were low in some surveillance studies (Wang et al., 2020), sporadic reports of CZA-resistant strains have rapidly increased, regardless of whether the patient had prior exposure to CZA (Humphries et al., 2015; Shields et al., 2016; Niu et al., 2020; Sun et al., 2020). Acquired resistance to CZA in KPC producers has been reported to involve several mechanisms, which mainly include missense mutations, insertions or deletions in the omega-loop (amino acid positions 164–179) of the KPC-lactamase, for example, D179Y in KPC-3, resulting in enhanced affinity toward ceftazidime with concomitant reduced binding to avibactam (Giddins et al., 2018).

Here, we described the emergence of the novel KPC-74 variant conferring CZA resistance originating from an ST11 KPC-2-producing CRKP clone that is widespread in China.

## Materials and Methods

### Patient, Strain, and Antimicrobial Susceptibility

The patient was a 68-year-old male who had suffered from myocardial infarction and subsequently developed ventilator-associated pneumonia during the hospitalization period from January to April 2020. A CZA-resistant *K. pneumoniae* strain, designated KP55, was recovered from sputum samples after a 2-week treatment with CZA at a dose of 2.5 g three times per day.

Antimicrobial susceptibility to amoxicillin-clavulanic acid, cefepime, ceftazidime, ertapenem, imipenem, meropenem, amikacin, ciprofloxacin, tigecycline, colistin, and CZA was determined using the broth microdilution method according to the Clinical Laboratory Standards Institute (CLSI, 2020) guidelines. MICs for all agents except tigecycline and colistin were interpreted according to the CLSI guidelines, and those for tigecycline and colistin were interpreted according to the European Committee on Antimicrobial Susceptibility Testing (EUCAST) breakpoints for *Enterobacteriaceae*.^1^ *Escherichia coli* ATCC 25922 was used as a quality control. NG-Test Carba 5 (NG Biotech, Guipry, France) was used to determine the carbapenemase enzyme type.

### Whole Genome Sequencing and *in silico* Analysis

The genomic DNA of the *K. pneumoniae* KP55 strain was extracted and sequenced with a combination of the Illumina-HiSeq X-Ten platform (Illumina Inc., San Diego, United States) and the long-read sequencing MinION platform (Nanopore, Oxford, United Kingdom) to obtain the complete genome sequence. The raw data acquired from the Illumina and MinION platforms were hybrid assembled by using Unicycler 0.4.8 (Wick et al., 2017). The RAST website server was then used for genome annotation.^2^ The comparison between the plasmid pKP55-2 and reference plasmid p3_L39 was illustrated by using CGview (Grant and Stothard, 2008). Multilocus sequence typing (MLST), acquired resistance genes and the Inc-type plasmid of the strain were screened and determined by using the MLST 2.0 server, ResFinder 4.0, and PlasmidFinder 1.3 at the Center for Genomic Epidemiology,^3^ respectively.

The crystal structure of KPC-2 (PDB 5UJ3) was retrieved from the Protein Data Bank and analyzed by the PyMOL Molecular Graphs System.^4^ KPC-2 is shown as a backbone ribbon, and the catalytic residue is shown in stick format. Important segments are highlighted by different colors.

### *bla*_KPC–74_-Harboring Plasmid Manipulations and *bla*_KPC_ Gene Cloning

The plasmid size was characterized via S1-nuclease pulsed-field gel electrophoresis (S1-PFGE), and the location of *bla*_KPC–74_ in the plasmid was determined by Southern blot hybridization between the S1-PFGE band and the *bla*_KPC–74_ gene probe. *Salmonella enterica* serotype Braenderup H9812 was used as the size marker (Hunter et al., 2005).

Plasmid extraction and transformation were performed to evaluate the resistance level mediated by the *bla*_KPC–74_-harboring plasmid according to the protocol from our previous study (Quan et al., 2017). Briefly, plasmid DNA was extracted using a Qiagen Plasmid Midi Kit (Qiagen, Hilden, Germany) and then electrotransformed into *E. coli* DH5α. The *bla*_KPC–74_-harboring plasmid transformants were selected on Mueller-Hinton (MH) agar plates containing ampicillin (100 mg/L) and were verified by *bla*_KPC–74_ gene PCR and sequencing. The MICs of the antimicrobial agents mentioned above for positive transformants were subsequently assessed.

The *bla*_KPC–74_ gene was also cloned to verify CZA resistance. The *bla*_KPC–74_ gene and wild-type *bla*_KPC–2_ gene, both containing the promoter region, were amplified from KP55 and a CRKP isolate from our previous study, respectively (Zhang et al., 2020). The purified *bla*_KPC_ PCR products were cloned into the pCR2.1-TOPO vector (Invitrogen, Shanghai, China), producing the recombinant plasmid pKP55 or pKPC-2. The recombinant plasmids were then introduced into the *E. coli* DH5α strain via chemical transformation experiments. Transformants were selected on MH agar plates containing 50 mg/L kanamycin and verified by *bla*_KPC_ gene PCR and Sanger sequencing.

### KPC ß-Lactamase Purification, Steady-State Enzyme Kinetic Measurements and Determination of IC_50_ Values

The *bla*_KPC_ gene sequence (residues 25–293) was cloned into the pET-28a vector to express an N-terminal His-tagged fusion protein. The vector was transformed into *E. coli* BL21 (DE3) competent cells and purified by nickel affinity chromatography and gel filtration (Pemberton et al., 2017). Protein concentration was determined by measuring the absorbance at 280 nm using an extinction coefficient of 39,545 M^–1^ cm^–1^, and the protein was then concentrated to 10–20 mg/mL.

Kinetic parameters were determined by using a spectrophotometer at room temperature and the purified enzymes. Each assay was performed in PBS at pH 7.4, and the data were fitted to the Michaelis-Menten equation to obtain the *K*_*m*_ and *k*_*cat*_ (Winkler et al., 2015). For nitrocefin, the values of *K*_*m*_ and *k*_*cat*_ were determined by measuring the initial velocities at a variety of nitrocefin concentrations, and substrate cleavage was monitored at 482 nm. For evaluation of the kinetic parameters of ceftazidime, the enzymes were mixed with various concentrations of the substrate, and the level of substrate cleavage at room temperature was monitored at 257 nm. The initial rates of ceftazidime cleavage were calculated. Similarly, the kinetic parameters of meropenem were calculated using the same protocol, but substrate cleavage was monitored at 297 nm.

The IC_50_ values for inhibition of the wild-type KPC-2 and KPC-74 proteins by avibactam, tazobactam and clavulanic acid were determined with nitrocefin as the substrate. The enzymes were mixed with these inhibitors at concentrations varying from 0 to 30 μM in PBS and incubated for 10 min, and 100 μM nitrocefin was subsequently added. The absorbance at 482 nm was recorded after 30 min and analyzed with Prism software (Tsivkovski and Lomovskaya, 2020).

### Ethical Approval

This study was reviewed and approved by the Research Ethics Committee of Zhejiang Provincial People’s Hospital (ref#2020QT308).

### Nucleotide Sequence Accession Numbers

The nucleotide sequences of *K. pneumoniae* KP55 reported in this study have been deposited in the GenBank nucleotide database under accession Nos. CP055294-CP055301. In particular, the *bla*_KPC–74_ sequence was deposited in the NCBI database under accession no. MT856045.

## Results

### Multidrug-Resistant *K. pneumoniae* Strain

**FIGURE 1:**
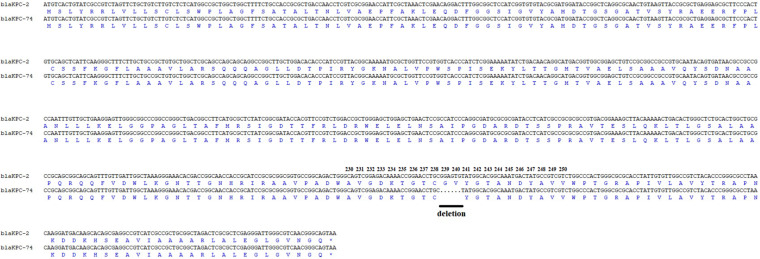
Amplicon alignments between *bla*_KPC–74_ and *bla*_KPC–2_ in nucleotide and amino acid (aa) sequences surrounding the mutation. Six nucleotide deletions were identified in the *bla*_KPC–74_ gene when compared to *bla*_KPC–2_, which led to amino acid deletions at the 239 and 240 amino acid positions in the KPC-2 protein. Black line: deletion.

*K. pneumoniae* KP55 was resistant to ertapenem (MIC, 16 mg/L), cefepime, ceftazidime, amoxicillin-clavulanic acid, amikacin, ciprofloxacin, and tigecycline but susceptible to imipenem (MIC, 0.5 mg/L), meropenem (MIC, 1 mg/L) and colistin (Table 1). The isolate was also resistant to CZA (MIC, 128 mg/L). The whole-genome sequencing data revealed that the *K. pneumoniae* KP55 strain belonged to ST11, which is the predominant clonal lineage of CRKP spreading in China, and clarified the encoded resistance genes, including *bla*_TEM–1_, *bla*_SHV–12_, *bla*_CTX–M–65_ *aadA2*, *rmtb*, *dfrA14*, *qnrS1*, *catA2*, *fosA*, *tet(A)*, *sul2* and a novel *bla*_KPC_ variant. The novel *bla*_KPC_ variant encoded by KP55 showed a deletion of 6 nucleotides at positions 712–717 compared to *bla*_KPC–2_, which led to it being designated *bla*_KPC–74_, and this deletion resulted in a variant enzyme with consequent deletion of glycine and valine at positions 239 and 240 (Figure 1).

**TABLE 1:** Antimicrobial susceptibility of the strains used in this study (mg/L).

Strains	AMC	FEP	CAZ	ETP	IPM	MEM	AMK	CIP	TGC	CST	CZA
*E. coli* DH5α	8	0.06	0.25	0.008	0.25	0.03	2	0.015	0.25	0.125	0.25
*E. coli* DH5α/pCR2.1	32	0.5	1	0.015	0.5	0.03	2	0.002	0.06	0.03	0.25
*E. coli* DH5α/pKPC-2	128	>128	64	64	64	16	4	0.004	0.06	0.125	0.25
*E. coli* DH5α/pKPC-74	64	4	128	0.03	0.25	0.015	4	0.002	0.06	0.125	16
*K. pneumoniae* KP55	64	256	>256	16	0.5	1	>256	>32	8	0.06	128
*E. coli* DH5α/pKP55_2	16	16	128	0.03	0.5	0.06	>256	0.015	0.06	0.03	16
*E. coli* ATCC 25922	8	0.06	0.125	0.008	0.125	0.015	0.5	0.125	0.03	0.25	0.25

*Avibactam was added at 4 mg/L. AMC, amoxicillin-clavulanic acid; FEP, cefepime; CAZ, ceftazidime; ETP, ertapenem; IPM, imipenem; MEM, meropenem; AMK, amikacin; CIP, ciprofloxacin; TGC, tigecycline; CST, colistin; CZA, ceftazidime-avibactam. E. coli DH5α/pCR2.1, E. coli DH5α was transformed by expression plasmid pCR2.1-TOPO as a control. E. coli DH5α/pKPC-2, E. coli DH5α was transformed by pKPC-2 plasmid carrying wild type bla_KPC–2_ gene. E. coli DH5α/pKPC-74, E. coli DH5α was transformed by pKPC-74 plasmid carrying bla_KPC–74_ gene. E. coli DH5α/pKP55_2, E. coli DH5α was transformed by wild type plasmid carrying bla_KPC–74_ gene from K. pneumoniae KP55.*

### *bla*_KPC–74_-Harboring Plasmid

**FIGURE 2:**
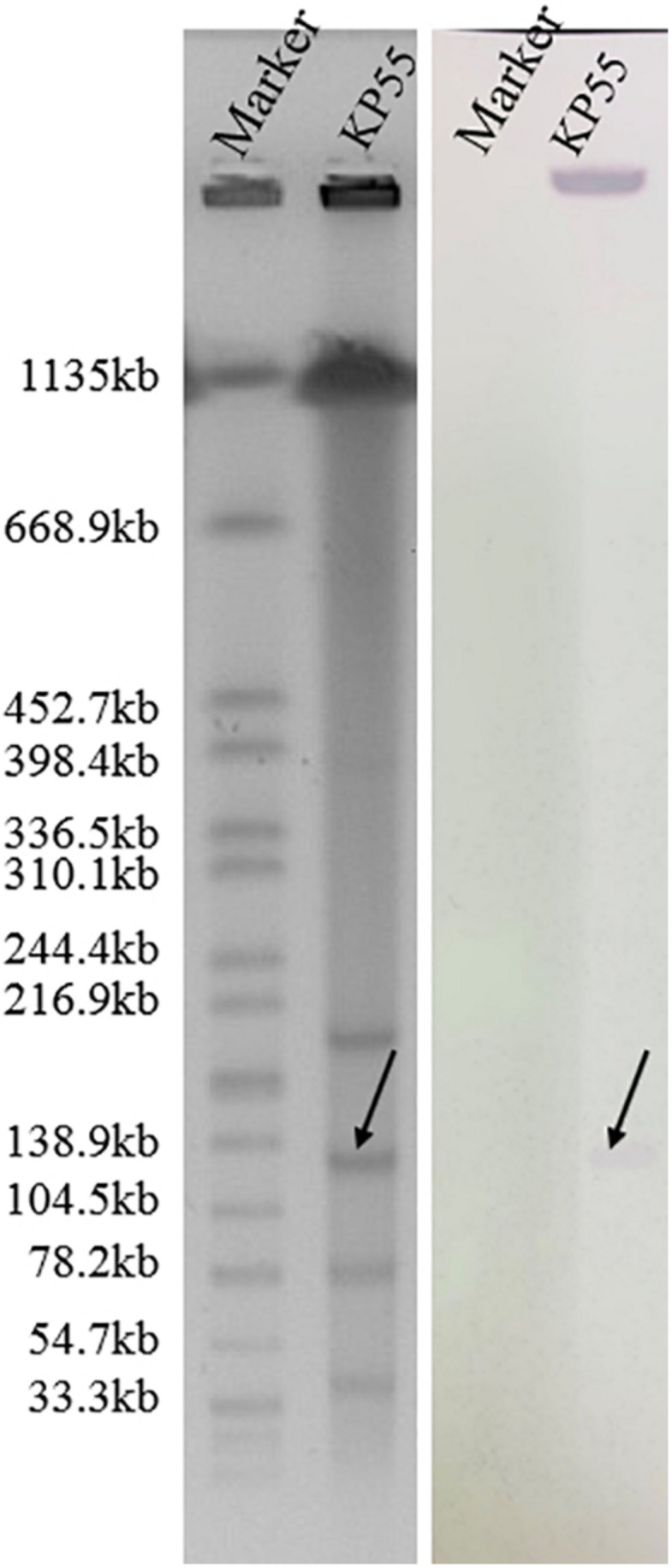
S1-digested plasmid DNA and Southern blot hybridization of the *bla*_KPC–74_-positive isolate. The bands indicated by arrows represent positive signals in Southern blot hybridization with the *bla*_KPC_ probe. The molecular marker *Salmonella* serotype Braenderup strain H9812 was used as the size marker.

**FIGURE 3:**
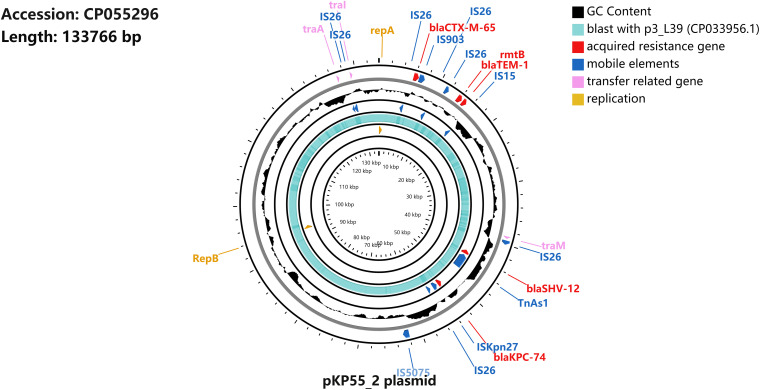
Schematic map of plasmid pKP55_2. The sequence alignment between pKP55_2 (accession number CP055296) and p3_L39 (accession number CP033956.1) is shown in the circle in light blue, and the GC content is shown in the circle in black.

S1-PFGE followed by Southern blotting showed that the *bla*_KPC–74_ gene was located on a plasmid (∼130 kb) (Figure 2). DH5α/pKP55_2 transformants harboring the *bla*_KPC–74_ gene exhibited a CZA MIC of 16/4 mg/L (a 64-fold increase compared with that of *E. coli* DH5α), indicating that the plasmid was able to transfer the CZA resistance phenotype to the recipient strain (Table 1). In addition, the complete plasmid sequence was obtained to characterize the genetic background of the CZA resistance plasmid, which was 133,766 bp in length with a GC content of 53.3% (Figure 3). The plasmid carried dual replicons, including IncFII (pHN7A8) and IncR. The mutant *bla*_KPC–74_ gene was flanked by IS*26* and Tn*As1* and presented a typical Tn*As1*-IS*Kpn6*-like-*bla*_KPC–74_-IS*Kpn27*-Tn*iA*-IS*26* genetic structure. Several antimicrobial resistance genes, such as *bla*_TEM–1_, *bla*_SHV–12_, *bla*_CTX–M–65_ and *rmtB*, were also detected in the same plasmid. Further sequence alignments revealed that the plasmid nucleotide sequences were identical (100% coverage and 99.99% identity) to that of the p3_L39 plasmid (accession number CP033956), which harbors *bla*_KPC–2_ and is carried by a CRKP strain found in China.

### The Novel Enzyme KPC-74 Is Responsible for CZA Resistance but Restores Susceptibility to Carbapenems

The CZA resistance phenotype was also confirmed by *bla*_KPC_ gene cloning and expression in *E. coli* DH5α. The clone carrying the wild-type *bla*_KPC–2_ gene in the pCR2.1-TOPO vector exhibited resistance to multiple β-lactams, including meropenem, imipenem and ertapenem, but susceptibility to CZA (0.25/4 mg/L). In contrast, the clone harboring the *bla*_KPC–74_-containing pCR2.1-TOPO plasmid exhibited resistance to CZA (16/4 mg/L) but increased susceptibility to meropenem, imipenem and ertapenem (Table 1), demonstrating that the *bla*_KPC–74_ gene was able to confer resistance to CZA (64-fold increase in MIC value) and restore susceptibility to carbapenems compared with *bla*_KPC–2_.

### Enzyme Kinetic Parameters and IC_50_ Values

The enzyme kinetics parameters of carbapenemase KPC-2 and its variant KPC-74 were subsequently compared to facilitate the understanding of the mechanism of CZA resistance. The apparent *k*_*cat*_/*K*_*m*_ of the KPC-74 enzyme with ceftazidime was twofold higher than that of wild-type KPC-2, and in particular, this enzyme exhibited a lower (∼9-fold) *K*_*m*_ with ceftazidime, indicating that the KPC-74 enzyme showed increased hydrolysis of ceftazidime compared with wild-type KPC-2 (Table 2). In contrast, KPC-74 exhibited decreased hydrolysis of nitrocefin compared with KPC-2, showing an ∼7-fold lower *k*_*cat*_/*K*_*m*_. Unexpectedly, the *k*_*cat*_/*K*_*m*_ of the KPC-74 enzyme for meropenem could not be measured by our methodology, although the KPC-2 *k*_*cat*_/*K*_*m*_ value for meropenem was ∼46-fold higher than that for ceftazidime (Table 2).

**TABLE 2:** Kinetic parameters of purified β-lactamases KPC-2 and KPC-74.

β-Lactam(s)	KPC-2				KPC-74		
	*K*_*m*_ (μM)	*k*_*cat*_ (s^−1^)	*k*_*cat*_/*K*_*m*_ (μM^−1^.s^−1^)		*K*_*m*_ (μM)	*k*_*cat*_ (s^−1^)	*k*_*cat*_/K_*m*_ (μM^−1^.s^−1^)
Nitrocefin	10	51	5.1		6.1	4.2	0.7
Ceftazidime	254	1.7	0.007		29	0.4	0.014
Meropenem	12	3.8	0.32		ND	ND	ND

**Inhibitor**							
	**IC_50_(μM)**							
			**KPC-2**		**KPC-74**		

Avibactam			0.027		0.454		
Tazobactam			1.63		0.034		
Clavulanic acid			0.764		0.043		

*Inhibitory concentration 50% (IC_50_) of β-lactamase inhibitors against KPC-74 and KPC-2 (bottom). ND: Not determinatable due to a low initial rate of hydrolysis. k_cat_, turnover; Km, Michaelis constant (Affinity); k_cat_/K_m_ = specificity constant (Hydrolysis). IC_50_ represents the concentration of a drug that is required for 50% inhibition of the enzymatic activity.*

In addition, the IC_50_ value, that is, the concentration for 50% inhibition, of avibactam was ∼17-fold higher against KPC-74 than against KPC-2, indicating that the G239_V240del observed in KPC-74 was associated with low affinity and consequent reduced sensitivity to avibactam. Conversely, the inhibitory activities of tazobactam and clavulanic acid against KPC-74 were higher than those against KPC-2, with ∼48- and 18-fold lower IC_50_ values, respectively (Table 2).

### KPC-74 Protein Modeling Analysis

**FIGURE 4:**
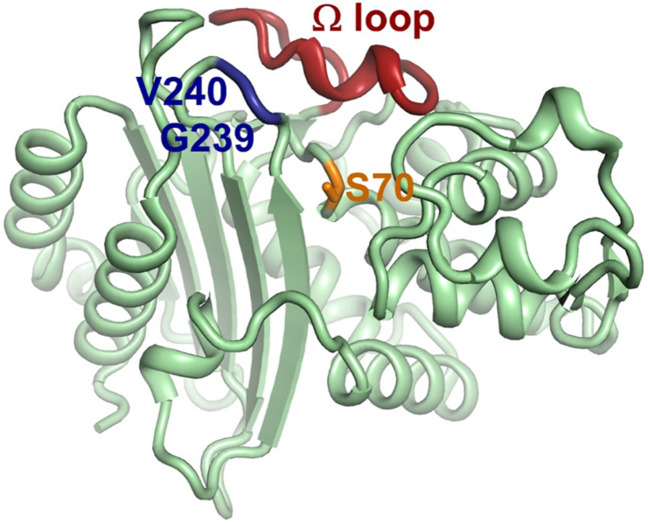
Crystal structure of KPC-2. The crystal structure of KPC-2 (PDB 5UJ3) was analyzed by the PyMOL Molecular Graphics System. KPC-2 is shown as a backbone ribbon. The catalytic nucleophile Ser70 is shown as sticks. G239_V240del is adjacent to the catalytic Ser70 and omega-loop according to the crystal structure.

Analysis of the crystal structure of the KPC-2 protein also revealed the potential influence of G239_V240del on the CZA resistance mechanism (Figure 4). The core of the active site of KPC carbapenemase contains a serine at position 70. Although G239_V240del observed in KPC-74 is distant from the catalytic nucleophile Ser70 in the primary structure, it is adjacent to Ser70 in the tertiary structure. G239_V240del would probably alter the local conformation and the hydrogen bond network of the active site, explaining the alterations in the substrate-binding profile and catalytic activity of KPC-74. Moreover, the residues G239 and V240 are also adjacent to the omega-loop in the tertiary structure.

## Discussion

Carbapenemase production is commonly responsible for CRKP worldwide, while the KPC-2 enzyme is the most frequently identified allele in China. The positivity rate of *bla*_KPC–2_ in CRKP isolates varies from 74 to 94% in different reports in China, but its spread is important, particularly because it is connected to the dominant clone, ST11 (Zhang et al., 2017; Wang et al., 2018; Zhang et al., 2018). The overwhelming majority of ST11-KPC-2 strains have become the most threatening clones and are the main strains of *K. pneumoniae* resistant to carbapenems in China. CZA exhibits good activity against KPC-2-type carbapenemase and has become the first-line option for the treatment of ST11-KPC-2 CRKP infection (Wang et al., 2020). However, the increasing reports of CZA resistance mediated by the mutated *bla*_KPC–2_ gene pose a great threat to clinical therapy, and limited therapeutic options are therefore available (Humphries et al., 2015; Shields et al., 2016; Niu et al., 2020; Sun et al., 2020).

Mutations of the *bla*_KPC–2_ gene in the omega-loop (amino acid positions 164–179), particularly at Ambler position 179, were the main factors responsible for CZA resistance (Livermore et al., 2015). Currently, more than 20 *bla*_KPC_ alleles that confer CZA resistance have been deposited in GenBank, and most of the mutations are in the omega-loop. In this study, however, the G239_V240del observed in KPC-74 was outside the omega-loop region of the KPC protein. Notably, amino acid substitutions, such as KPC-8 (Val240Gly and His274Tyr) (Gregory et al., 2010) and KPC-23 (Val240Ala and His274Tyr) (Galani et al., 2019), occurred outside the omega-loop region of KPC, but resistance to CZA has also been reported. In addition, a recent study found that CZA resistance in *K. pneumoniae* was involved in amino acid deletion at different positions of KPC-3 (Glu167_Leu168del), indicating that amino acid deletion of the KPC protein could be associated with CZA resistance (Antinori et al., 2020).

Both transformation of the *bla*_KPC–74_-harboring plasmid and the *bla*_KPC–74_ gene cloning assay confirmed the CZA resistance phenotype of the novel KPC variant, with a 64-fold increase in the CZA MIC compared with that toward the recipient strains. In terms of steady-state enzyme kinetic measurements, the data were fitted to the Michaelis-Menten equation to obtain *K*_*m*_ and *k*_*cat*_ values. The former represents the affinity between enzyme and substrate as a Michaelis constant, and the latter represents the turnover number of enzymes as the catalytic constant. Furthermore, *k*_*cat*_/*K*_*m*_ is the specificity constant, reflecting the hydrolytic capacity of the enzyme. The enzyme kinetic parameters and IC_50_ values further indicated that the hydrolytic activity of the KPC-74 enzyme against ceftazidime was potentiated twofold and that the affinity between KPC-74 and avibactam was alleviated 17-fold compared with that of the KPC-2 allele. The *bla*_KPC–74_ gene was able to restore susceptibility to carbapenems compared to *bla*_KPC–2_, suggesting a trade-off phenomenon in the resistance development process. The evolved variant KPC-74 was resistant to CZA due to enhanced hydrolysis of ceftazidime with a concomitant reduction in the binding affinity for avibactam; this was accompanied by reduced activity against carbapenems and increased inhibitory activities of classic inhibitors such as tazobactam and clavulanic acid, compared with the ancestral allele (KPC-2), which demonstrates diversity in resistance evolution.

A previous study posited that there are 4 loops surrounding the active site Ser70 that are possibly associated with CAZ resistance: the important omega-loop (Arg164 to Asp179), the loop between the α3- and α4-helices (Leu102 to Ser106), the loop between the β3- and β4-strands (Cys238 to Thr243), and the loop between the β5-strand and α11-helix (Ala267 to Ser275) (Levitt et al., 2012; Galdadas et al., 2018). G239_V240del was present in the loop 238–243.

The plasmid encoding the *bla*_KPC–74_ gene may also cause further dissemination of CZA-resistant bacteria. Furthermore, the plasmid carries dual replicons (*Inc*FII [pHN7A8] and *Inc*R). The presence of dual replicons may be beneficial to the maintenance, stable replication and broad host range of the strain with low fitness costs (Sekizuka et al., 2018). Therefore, effective measures must be immediately taken to prevent the spread of the resistant plasmid.

Notably, CARBA 5, a commercial rapid detection method for *bla*_KPC–2_-positive strains, showed negative results for strains producing KPC-74 (data not shown). G239_V240del of KPC-2 probably influenced the binding efficiency of the KPC-2 protein antibody and consequently resulted in the failure of the detection system. Currently, most CZA-resistant isolates are reported to be susceptible to meropenem and cannot be identified as KPC-producing strains (Shi et al., 2020), which leads to their misleading detection in clinical laboratories. Thus, it is imperative to improve the molecular screening method for *bla*_KPC_ to facilitate its rapid and accurate detection.

In summary, we described a novel KPC variant, KPC-74, with a mutation in the omega-loop region that conferred resistance to CZA in an ST11-type clinical CRKP isolate after CZA exposure. KPC-74 showed CZA resistance by exhibiting higher hydrolysis than wild-type KPC-2 toward ceftazidime and reduced affinity to avibactam but restored susceptibility to carbapenems. This CZA resistance caused by *bla*_KPC_ gene mutation could be selected after CZA therapy and evolved to be more diverse and heterogeneous. The surveillance of CZA resistance particularly in patients who accept CZA therapy is urgently needed in clinical settings.

## Data Availability Statement

The datasets presented in this study can be found in online repositories. The names of the repository/repositories and accession number(s) can be found in the article/supplementary material.

## Author Contributions

YJ and YY conceived and designed the experiments. XL and JQ performed the experiments. HK, YF, and WW analyzed the data. XL and YJ wrote the manuscript. All authors read and approved the final manuscript.

## Conflict of Interest

The authors declare that the research was conducted in the absence of any commercial or financial relationships that could be construed as a potential conflict of interest.

## Publisher’s Note

All claims expressed in this article are solely those of the authors and do not necessarily represent those of their affiliated organizations, or those of the publisher, the editors and the reviewers. Any product that may be evaluated in this article, or claim that may be made by its manufacturer, is not guaranteed or endorsed by the publisher.

## Footnotes

1
http://www.eucast.org/clinical_breakpoints

2
https://rast.nmpdr.org/

3
http://www.genomicepidemiology.org/

4
http://pymolwiki.org/PLoS
